# An Inspection Technique Using Fit Clearance Based on Microscopic Vision in Precision Assembly

**DOI:** 10.3390/mi14101852

**Published:** 2023-09-27

**Authors:** Yawei Li, Yi Luo, Xiaodong Wang

**Affiliations:** 1 Key Laboratory for Micro/Nano Technology and System of Liaoning Province, Dalian University of Technology, Dalian 116024, China; ginliyawei@163.com (Y.L.);; 2 Key Laboratory for Precision and Non-Traditional Machining of Ministry of Education, Dalian University of Technology, Dalian 116024, China

**Keywords:** inspection, microscopic vision, precision assembly, fit clearance

## Abstract

Inspection is a crucial process to ensure product quality. In the precision assembly of an optic-mechanical device, a part with micro multi-section arcs needs to be inspected and assembled into another part. Actually, because of machining errors, including dimensional and geometric shapes, can lead to complex deformation modes for parts with micro multi-section arcs, posing challenges to their inspection. Furthermore, inconsistencies in feature images in microscopic vision may complicate the extraction of the Region of Interest (ROI). To address these issues, this paper proposes an ROI extraction method based on the CAD model for rough positioning of feature points and connected region detection for refinement. Subsequently, based on feature points, the CAD model is used again to obtain the ROI. For inspection purposes, this paper proposes a method suitable for micro multi-section arcs based on assembly fit requirements. Experimental testing was performed on parts with eight-section arcs and mirrors to verify the effectiveness of the proposed method. This method provides a suitable solution for the inspection of micro multi-section arcs in precision assembly with the potential to improve the accuracy of the inspection results.

## 1. Introduction

Microscopic vision is commonly used for alignment in precision assembly and micromanipulation [1,2,3,4,5,6]. Additionally, inspection plays an important role in quality control as it directly affects the performance and reliability of the assembled products [7,8,9]. In an optic-mechanical device assembly, a part with micro multi-section arcs needs to be assembled into another part. They are bonded together using adhesive, and the thickness of the adhesive layer has a direct impact on its bonding strength. Therefore, it is crucial to strictly control the fit clearance between them. However, the parts consist of a micro, multi-section arc structure that may have machining errors, leading to the structural deformation of the arcs. This means that not all arc structures are perfectly circular, and a significant deformation may cause the part to fail to meet the assembly requirements. Hence, it is essential to inspect parts with micro multi-section arcs before assembly to ensure that they meet the necessary quality standards. Due to the deformation of the parts, only the dimensional information of the parts cannot be accurately inspected, and the parts need to be inspected according to the fit clearance of the assembly. For the machining deformation of micro multi-section arcs, the feature images in microscopic vision may contain inconsistent noise points and lack clear boundaries, making Region of Interest (ROI) extraction difficult. In addition, it brings a significant challenge in determining the proper center for the assembly alignment position.

There are several methods to extract ROI. These include image segmentation [10,11,12], histogram clustering [13,14], a fixed-area method [15,16], a template matching method [17,18], a golden template method [19,20], and a feature point method [21,22,23,24]. For the parts with micro multi-section arcs, due to machining errors, the background of feature images can differ due to structural deformation, and the shape of feature images can be irregular due to machining texture. In such cases, template matching may not be suitable since it only works for parallel movement and is not suitable for rotation or shape changes of the matching object in the image. Additionally, the fixed-area method cannot be used to extract ROI when the position of the feature image changes within the field of view. When dealing with different backgrounds and irregularly shaped images, segmentation and feature point extraction become challenging. Segmentation methods may require manual adjustment of parameters, making them less efficient and subject to human errors. Meanwhile, feature point extraction may be affected by the irregularity of the image shape, leading to less accurate extraction results. Therefore, it is necessary to propose an ROI extraction method suitable for the detection of micro multi-section arc structures.

For parts with an approximately circular structure, the assembly alignment position generally uses the center of the circle. Various methods can be used to calculate the center of a circle, including least squares fitting, Minimum Zone Circle, Maximum Inscribed Circle, Minimum Circumscribed Circle [25,26,27], and constrained least squares fitting [28]. There are also other methods, such as the Hough transform [29], randomized algorithm [30], and power histograms [31] that can determine the center of a circle from digital images. However, these methods are suitable for calculating the structure close to an ideal circle; they cannot accurately determine the center of parts with micro multi-section arcs because they deviate from an ideal circle. For actual parts, the machining of the shaft and hole is not always a standard cylindrical surface due to manufacturing precision, deformation, and other factors. To address this issue, Wang et al. [32] considered the weight of each geometric feature in the assembly accuracy index based on the actual assembly process and specific assembly objects. By obtaining a comprehensive evaluation index of component assembly accuracy, the optimal matching pose for assembly can be determined. Similarly, Wu et al. [33] used the model of maximizing the minimum clearance between the axle and hole to determine the optimal assembly position and pose for precise assembly operations of certain weapon equipment. This approach helps avoid collision and scraping during docking and improves the assembly success rate. In fact, uniform stress resulting from the assembly plays a critical role in the mechanical structure’s life and the component’s performance [34]. For instance, uniform bolt force is necessary to achieve a uniform distribution of gasket stress, ensuring leak-free service of the joint in the assembly of pressure vessel flanged connections [35]. Meanwhile, the uniform distribution of assembly features, such as form errors and stresses, significantly influences the accuracy and stability of precision instruments [36,37]. For the assembly of parts with micro multi-section arcs and other parts, poor uniformity in the thickness of the adhesive layer between parts can have a negative impact on the performance of optic-mechanical devices. Therefore, to ensure the thickness of the adhesive layer between parts is more uniform, the uniformity of the fit clearance areas in the predicted assembly is considered when determining the assembly position of parts with micro multi-section arcs.

This paper proposes an inspection method in microscopic visualization for parts with micro multi-section arcs. To extract the ROI of the parts’ images, we utilize a feature point extraction method that combines CAD-based rough position and connected region detection correction; after that, the CAD model is used again to obtain the ROI. For inspecting the part, we propose a novel method based on the assembly fit requirements. Specifically, we use the minimum variance of assembly fit clearance areas as the suitable assembly alignment position.

The remainder of this paper is organized as follows. Section 2 describes the experiments and tasks in this study. Section 3 presents an ROI extraction method for feature images, including rotation, shapes, and size inconsistencies. Section 4 illustrates the inspection method and the methodology for calculating the assembly alignment position for parts with eight-section arcs, and then gives the inspection results of three parts to verify the proposed method. Finally, Section 5 presents the conclusions of this study.

## 2. Assembly System and Task

**Figure 1:**
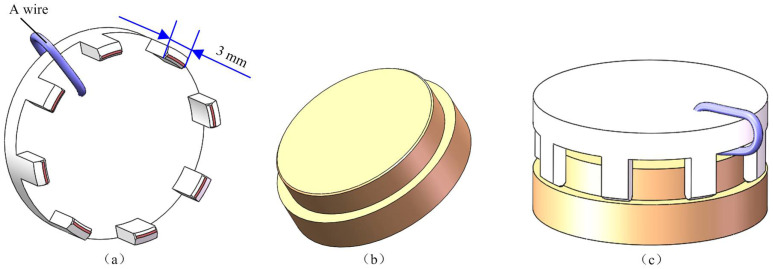
Parts and assembled component: (**a**) Claw framework; (**b**) Mirror; (**c**) Assembled component.

As illustrated in Figure 1, the assembly of an optic-mechanical device requires combining a claw framework as depicted in Figure 1a and a mirror as depicted in Figure 1b, forming the component shown in Figure 1c. To achieve the corresponding function, a wire is required to weld the outer and inner layers of the claw framework. However, to place a wire without compromising the assembly, it is necessary to cut out some sections of the claw framework. As a result, the framework is composed of multi-section arcs. Therefore, the framework can consist of three or more arc segments. In this study, we focus on the claw framework with micro eight-section arc structures as an example, with the length of each arc section being less than 3 mm.

In the assembly, first, the size of the arcs of the claw framework must be inspected, and then, the inner arcs are manually coated with glue; after that, it is assembled on the mirror using the mechanical arm of the precision assembly system. Because the two parts were assembled using adhesive bonding, the thickness of the adhesive layer must be within a certain range to ensure optimal bonding strength. Therefore, the fit clearance value between the two parts must be carefully controlled. Although the mirror machining accuracy is high, errors in the claw framework machining can cause deformation of the arc structures. As a result, inspecting this part is of great significance for ensuring the assembly quality of this optic-mechanical device.

**Figure 2:**
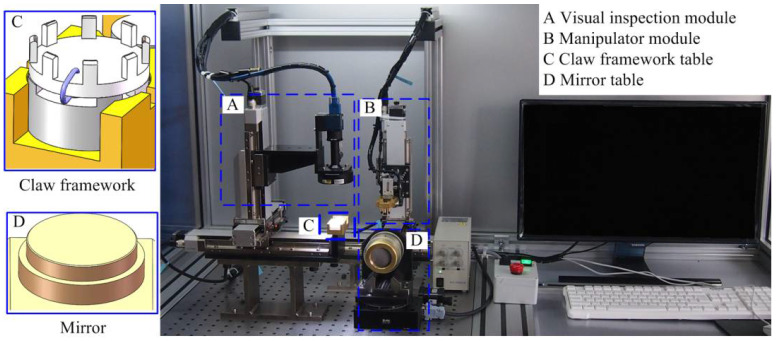
Assembly system.

The assembly system is depicted in Figure 2. It comprises a visual inspection module, a manipulator module, a claw framework table, and a mirror table. The visual inspection module consists of a visual component and an X-Y-Z precision stage. It includes a camera, a telecentric lens, a coaxial light source, and a ring light source, which is aimed at inspecting the claw framework and assisting with the alignment of the assembly. It used a camera with a resolution ratio of 3088 × 2064 and a pixel size of 2.46 μm × 2.46 μm, as well as a telecentric lens with a magnification of 1 to obtain high-resolution images of the parts. The manipulator module is used to pick up the claw framework and realize the assembly with the mirror. Tables for the claw framework and mirror were used to position the parts accurately.

**Figure 3:**
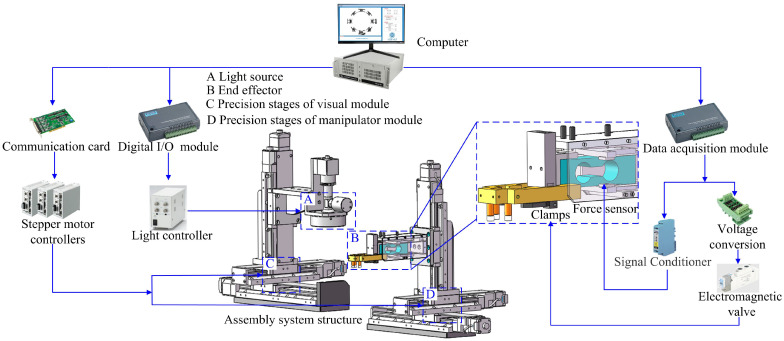
Hardware relationships of assemble system.

The hardware relationships of the assembly system can be established based on its structure, as shown in Figure 3. The 3-DOF precision stages of the visual module and manipulator module are connected to an Industrial Personal Computer (IPC) via motion controllers and a communication card to realize displacement in the *X*, *Y*, and *Z* directions. The light controller communicates with the IPC using a digital I/O module to control the brightness of coaxial and ring light sources. A force sensor and clamp are installed on the end effector of the manipulator module. The force sensor is connected to the IPC via a signal conditioning module and a data acquisition module to achieve accurate control of the end effector in the *Z* direction. The clamp is mounted on the cylinder, which is connected to the electromagnetic valve and voltage conversion module, enabling the operation of clamping and releasing of the claw framework. Utilizing this experimental system it ensures the precise assembly of the parts and compliance with the relevant assembly requirements.

To obtain high-resolution images, the field of view is small. When the size of the parts is larger than the field of view, the visual module acquires partial images of a part, and the entire image is obtained using image mosaic due to the precision stages with high repeatable positioning accuracy in precision assembly, which was 0.5 μm. Therefore, to reduce the workload, an image mosaic of parts is performed based on the performance of the hardware.

The transformation model between two images captured at different positions in the system can be described as follows:

(1) $$\left[\begin{array}{c} x\\ y \end{array}\right]=\left[\begin{array}{c} x_{0}\\ y_{0} \end{array}\right]+\left[\begin{array}{c} \cos\alpha \\ \sin\alpha \end{array}\right]dx+\left[\begin{array}{c} \sin\beta \\ \cos\beta \end{array}\right]dy$$

where (*x*_0_,*y*_0_) represents the coordinate point of the image acquired at the initial position, (*x*,*y*) represents the coordinate point of the image acquired when the visual device moves distances *dx* and *dy* in the *X* and *Y* directions, respectively, and *dx* and *dy* are positive when the motion direction is along the positive direction of the coordinate axis. *α* and *β* signify the installation angles of the visual device in the *X* and *Y* directions, respectively. The angle was calibrated using a calibration plate with an accuracy of 1 μm. Using this relationship, the stitched image could be obtained.

**Figure 4:**
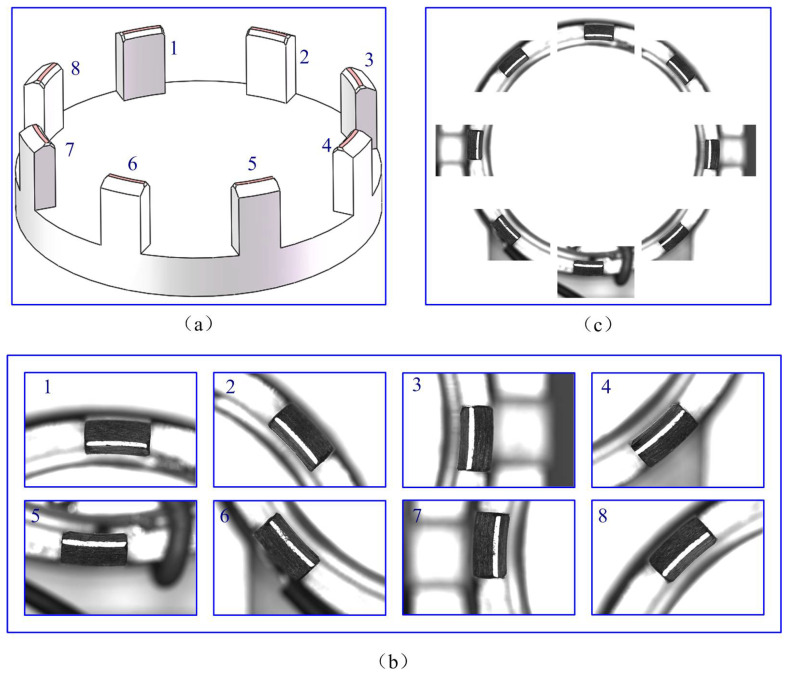
Experimental image of a claw framework: (**a**) Structure number; (**b**) Partial images; (**c**) The mosaic image.

For the experiment of the claw framework, the images were captured under identical lighting conditions. As depicted in Figure 4, it shows an experimental image of a claw framework. For convenience in description, the arcs of the eight-section are numbered 1–8 in a clockwise manner, as shown in Figure 4a. The partial images and the entire image are shown in Figure 4b,c, respectively. 

**Figure 5:**
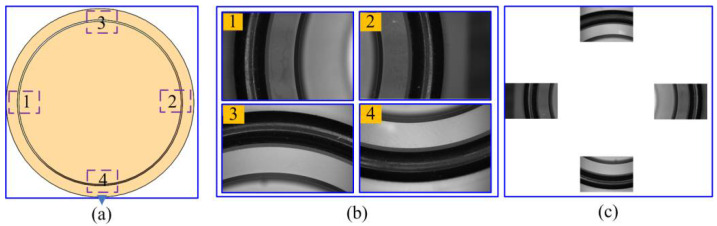
Experimental image of a mirror: (**a**) Image acquisition position; (**b**) Partial images; (**c**) the mosaic image.

Figure 5 shows an experimental image of a mirror, which is obtained using the image mosaic technique, similar to the claw framework. The image acquisition position is depicted in Figure 5a. Because a unique circle can be determined using two points passing through the center, at least two arc segments passing through the center of the circle are required to determine the size of the part. Meanwhile, the more the image information, the more accurate the measurement result. When measuring the mirror, it is easy to obtain images 1, 2, 3, or 4 in Figure 5a, only moving the *X*- or *Y*-axis of the visual system. To use more image information and reduce the workload of image acquisition, the information of these four groups of arc segments was used to calculate its size. The partial images and the entire image are shown in Figure 5b,c, respectively (In practice, a black rubber ring is installed on the outer ring of the mirror to change the contrast of the image, making it easier to extract edges).

By utilizing this approach, we can obtain high-resolution images of the assembly parts, enabling the accurate evaluation of fit clearance values and the determination of the optimal alignment position for assembly.

## 3. ROI Extraction

### 3.1. Feature Image of Micro Multi-Section Arcs

**Figure 6:**
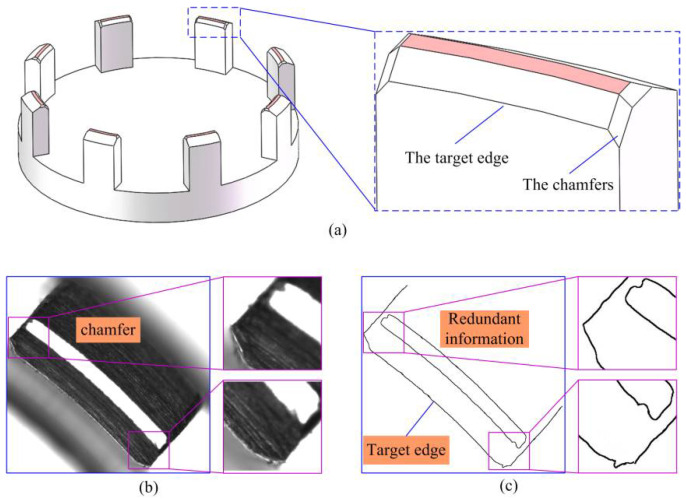
The target edge with the redundant chamfers information: (**a**) The chamfers of arc structure; (**b**) The chamfers image; (**c**) Redundant information between the target edge.

Because the inner arc of the claw framework needs to be matched with the mirror to form the component of the optic-mechanical device, the thickness of the adhesive layer between it and the mirror needs to be controlled within a certain range to ensure the strength of the bond. Therefore, for the part with eight-section arcs, the inner arc is the key feature that requires measurement. We refer to this as the target edge, which must be obtained accurately. However, there are chamfers on both sides of the inner arc, as depicted in Figure 6a. This results in the target edge image containing redundant chamfer information on both sides, as shown in Figure 6b,c. Therefore, it is necessary to extract the ROI and then obtain the target edge from it.

**Figure 7:**
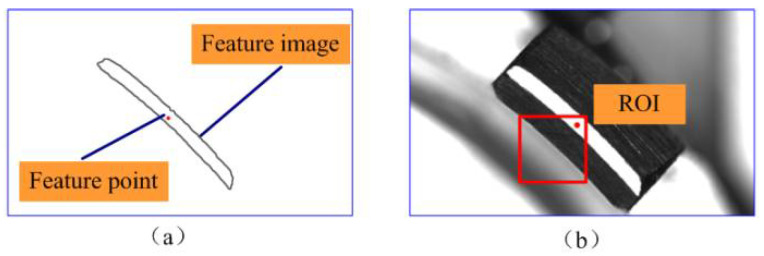
Feature point and ROI: (**a**) Feature image and feature point; (**b**) The ROI of the image of one arc structure.

Based on the characteristics of the image, a closed contour can be considered a feature image, and its centroid can be obtained after processing, as shown in Figure 7a. The centroid serves as the feature point of the feature image. Next, the ROI of the image can be described as a rectangular area, as depicted in Figure 7b, based on the feature point of the feature image and the design dimensions of the part. Therefore, it is only to extract the feature point of the feature image, and the ROI of the image can be obtained using the feature point.

**Figure 8:**
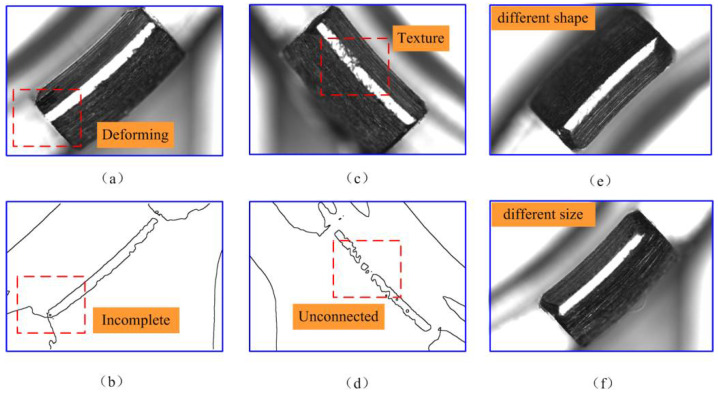
Structural deformation and processing texture of the feature image: (**a**) The deforming part; (**b**) The image preprocess result of the deforming part; (**c**) The part with machining texture; (**d**) The image preprocess result of the part with machining texture. (**e**) The feature image has a different shape. (**f**) The feature image has a different size.

Actually, this part has manufacturing errors, in which processing deformation and texture cannot be avoided in some of the structures, leading to their feature images being non-identical under the same lighting conditions. For instance, as shown in Figure 8, some of the feature images exhibit structural deformation or processing texture. In Figure 8a, the arc structure has been deformed by machining or stressing, and there is no clear boundary between the feature image and the background. Therefore, the outline of the feature image is an incomplete region, as shown in Figure 8b. In addition, Figure 8c shows that the feature image has noise points due to the texture created by machining, and its outline of the feature image is unconnected, as shown in Figure 8d. In image processing, the contour extraction method is used to extract the contour of the feature image, and then the centroid can be extracted. Therefore, the outline of the feature image must be complete and connected. However, it is challenging to extract the complete contour for all images.

Moreover, the size and shape of the feature images are also inconsistent, as depicted in Figure 8e,f. Therefore, the feature images of this part exhibit rotational changes, inconsistencies in size and shape, the presence of noise, and a lack of clear boundaries in some cases. Consequently, it is challenging to extract all features accurately from the image.

Despite these difficulties, it is crucial to obtain accurate feature images of parts with micro multi-section arcs to ensure the accuracy of the measurement process. Further research is necessary to address these challenges and develop effective methods for extracting features in precision assembly.

### 3.2. ROI Extraction Based on CAD Model

Although it is difficult to extract all feature points from all images, feature extraction is carried out on one part image at a time during actual measurement, in which only eight feature points need to be extracted per measurement. Generally, after adjusting the appropriate image processing parameters, at least one feature point can be obtained from an image of a part directly. In fact, for a mechanical part whose CAD models are typically known, there exists a geometric relationship among feature points. Based on this relationship, images of a part can be divided into two sections: those from which feature points can be extracted directly and those that cannot. The relationship between the feature points in these two sections can be described as follows.

(2) $$\left[\begin{array}{c} {\hat{x}}_{i}\\ {\hat{y}}_{i} \end{array}\right]=\left[\begin{array}{c} {\bar{x}}_{i}\\ {\bar{y}}_{i} \end{array}\right]+\left[\begin{array}{c} f_{ix}\\ f_{iy} \end{array}\right]i=1,2,\ldots ,8$$

where $\left({\bar{x}}_{i},{\bar{y}}_{i}\right)$ represents the coordinates of the feature points that can be directly extracted from the feature images, $\left({\hat{x}}_{i},{\hat{y}}_{i}\right)$ represents the coordinates of the others that cannot, *f_ix_* and *f_iy_* represent the geometric relationships between the feature points of two feature images in the *x* and *y* directions, and *i* represents the serial number of feature image.

For feature images from which feature points cannot be directly obtained using adjusted processing parameters, their approximate feature points can be calculated using the following formula:

(3) $$\left[\begin{array}{c} {\tilde{x}}_{i}\\ {\tilde{y}}_{i} \end{array}\right]=\left[\begin{array}{c} \sum_{i=1}^{n}{\hat{x}}_{i}/n\\ \sum_{i=1}^{n}{\hat{y}}_{i}/n \end{array}\right]i=1,2,\ldots ,8$$

where $\left({\tilde{x}}_{i},{\tilde{y}}_{i}\right)$ represents the approximate feature points of the image.

**Figure 9:**
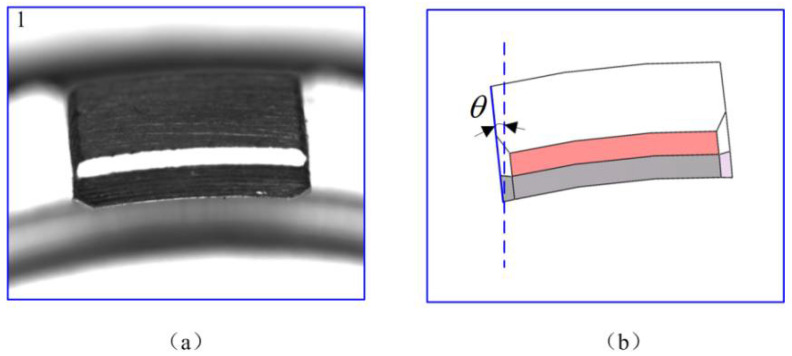
Deflection angle relative to the ideal position: (**a**) Image of the No.1 arc structure; (**b**) The rotation angle of the structure.

For the image of this part, as shown in Figure 9a, the arc structure is viewed at a certain angle. Therefore, to establish the relationship between feature points, it is necessary to calculate the deflection angle relative to the ideal position. Considering the structural characteristics, the deflection angle of the No. 1 arc structure equals the actual deflection angle of the part. As shown in Figure 9b, the angle of rotation *θ* can be obtained by calculating the slope of the left edge.

Based on the deflection angle, a geometric model of the feature points can be established. According to the CAD model, the feature points are situated at the eight vertices of a regular octagon. For a given part, the relationship between its eight feature points can be obtained using the CAD model and deflection angle. In feature extraction, parameters suitable for general images are first adjusted, and then feature extraction is carried out on one part’s image. The feature points that cannot be directly extracted from the approximate positions can be determined based on the extracted feature points and the known relationship between them. However, due to machining errors, there may be differences between actual parts and CAD models, which means that this method only yields approximate feature point positions. To obtain the accurate positions of feature points, the approximate positions are used as prior information. Then, the accurate feature point positions can be obtained by detecting the centroid position of the connected regions and comparing them with the approximate positions. For images with feature points obtained by the CAD model, the centroid positions of multiple connected regions in the image are extracted (The connected region extraction function in Opencv 3 was used, and the filter set smaller parameters) and compared with the rough position, where the centroid position with the smallest distance from the approximate position is the accurate position.

**Figure 10:**
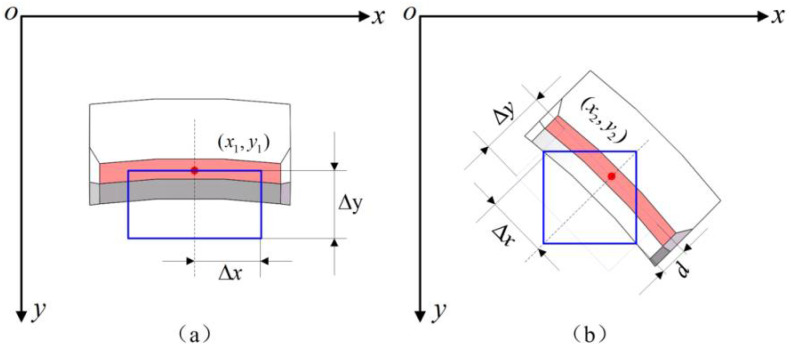
The relationship between feature point and ROI: (**a**) The ROI of the vertical direction arc structure; (**b**) The ROI of the oblique direction arc structure.

The ROI of the image can be obtained from its feature points using the CAD model again. The origin coordinates of the ROI can be expressed as:

(4) $$\left[\begin{array}{c} X_{i}\\ Y_{i} \end{array}\right]=\left[\begin{array}{c} x_{i}\\ y_{i} \end{array}\right]+\left[\begin{array}{c} g_{ix}\\ g_{iy} \end{array}\right],\;i=1,2,3,\cdots \cdots ,8$$

(5) $$\left[\begin{array}{c} X_{i}^{\prime }\\ Y_{i}^{\prime } \end{array}\right]=\left[\begin{array}{c} x_{i}\\ y_{i} \end{array}\right]+\left[\begin{array}{c} h_{ix}\\ h_{iy} \end{array}\right],\;i=1,2,3,\cdots \cdots ,8$$

where (*X*,*Y*) and (*X*’,*Y*’) represent the top-left and bottom-right vertex coordinates of the target region, (*x_i_*, *y_i_*) represents the feature point coordinates of the ROI, *g_ix_*, and *g_iy_* represent the relationship between a vertex and feature points, and *h_ix_* and *h_iy_* represent the relationship between another vertex and feature points, respectively. By using the designed dimensions as a reference and defining a rectangular area, we can establish the relationships between the feature points and the rectangular region in the vertical and oblique directions, as shown in Figure 10a,b, respectively. Assume that Δ*x* and Δ*y* are the values that are set in light of the designed dimensions, and *d* represents the distance from the feature point to the tangent line of the inner arc. The origin coordinates of the ROI can be obtained from the relationships Δ*x*, Δ*y*, and *d*, which are listed in Table 1.

**Figure 11:**
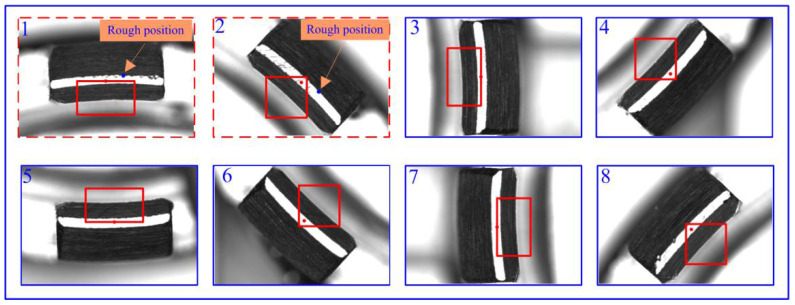
ROI extraction image of a part.

The ROI extraction result of a typical part is shown in Figure 11. It is worth noting that the feature images of arc segments 1 and 2 cannot directly extract feature points due to noise. However, their feature points can be obtained by first acquiring their rough position from the CAD model and then refining it using the method of connected region detection.

Overall, the proposed method, which combines CAD-based rough position and connected region detection correction, provides an effective approach to address the issues associated with the extraction of ROI from images of parts with micro multi-section arcs.

After extracting the ROI of the part, its target edge can be obtained, and the part can be inspected.

## 4. Inspection

### 4.1. The Inspection Principle

Machining errors in the claw framework can lead to the deformation of the structure, which can compromise the quality and reliability of the optic device. Consequently, it is essential to inspect this part thoroughly before assembling it.

**Figure 12:**
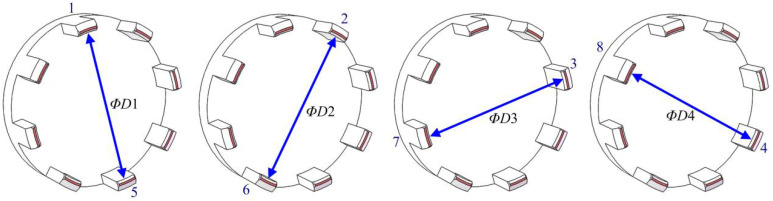
Diameters of arcs in opposite directions.

As illustrated in Figure 12, *ΦD*_1_, *ΦD*_2_, *ΦD*_3_, and *ΦD*_4_ represent the diameters of the arcs in the opposite direction. In manual inspection, a vernier caliper was typically used to measure the dimensions of the arcs in opposite directions to determine whether they met the assembly requirements. However, due to arc deformation, even if the four grouping diameters meet, the parts may not satisfy the assembly requirements. For instance, when the arcs of Nos. 1 and 5 have large deformations in the same direction, the part cannot be assembled with the mirror, even though the diameter of *ΦD*_1_ meets the design requirement. Therefore, only the dimensions of the arcs in opposite directions can easily lead to false detection.

Due to the fact that this part is composed of eight segmented arcs, there are various potential conditions for deformation. Thus, it becomes very complex to analyze the deformation directly. In manual inspection, only the size of the structures can be obtained for micro multi-section arcs. However, in precision assembly, not only can dimensional information of two parts to be assembled be obtained but also the values of predictive assembly fit clearance between the two parts can be calculated. Actually, the measurement and control of the fit clearance are of great significance to guarantee the assembly accuracy and reliability of the parts in precision assembly [38,39]. Because the thickness of the adhesive layer between two assembled parts needs to be controlled within a certain range, the fit clearance value also needs to be within a specific range. Therefore, the inspection of micro multi-section arcs can be performed according to their fit clearance values.

**Figure 13:**
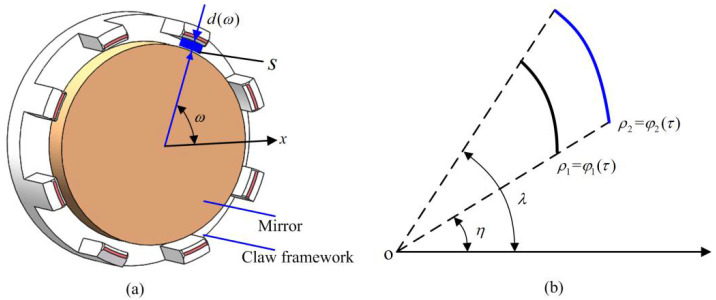
Fit clearance values: (**a**) Fit clearance value about claw framework and mirror; (**b**) Arcs in polar coordinates.

According to the measurement data of the claw framework and mirror, the fit clearance values of the predictive assembly can be calculated. These values can be used to determine whether the claw framework meets the assembly requirements. These values are denoted as *d*_1_*(ω)*, *d*_2_*(ω)*,…, *d*_7_*(ω)*, and *d*_8_*(ω)* in Figure 13a, where *ω* represents the angle between the points of the arcs and the *X*-axis.

To facilitate the calculation, the data for the mirror and eight-section arcs were converted to polar coordinates. As shown in Figure 13b, the functions of the two parts are described as *ρ*_1_ *= φ*_1_(*τ*) and *ρ*_2_ *= φ*_2_(*τ*) in polar coordinates, and their angles with respect to the polar axis are *η* and *λ*, respectively. Thus, the fit clearance value between an arc of the claw framework and a mirror is:

(6) $$d_{i}=\varphi_{i2}(\tau )-\varphi_{i1}(\tau )i=1,2,\ldots ,8$$

According to the above formula, the mean fit clearance values of each arc with the mirror can be obtained, and these values can be used to inspect the claw framework.

To calculate the fit clearance values for the assembly of the claw framework and mirror, it is important to know the assembly alignment position of the assembled parts. The mirror has high machining accuracy, so its center can be calculated using the least squares fitting method. However, for the claw framework, due to machining deformation, the arc segments of the part are not on an ideal circle. Therefore, the center of the micro multi-section arcs calculated by the least squares fitting method is not a suitable alignment position for its assembly. If the claw framework is assembled with this center, the consistency of the assembly fit clearance between each arc section and mirror will be poor. This will produce uneven assembly force and affect the stability of optic-mechanical devices. Thus, to perform predictive assembly, the assembly alignment position of the parts with micro multi-section arcs must be thoroughly studied.

### 4.2. Assembly Alignment Position

To solve the problem of assembly alignment position of parts with micro multi-section arcs, a method based on uniformity assembly was presented. Because the claw framework and the other part were assembled by adhesive bonding, to make the thickness of the adhesive layer between them more uniform, the fit clearance areas should be made uniform.

Due to the deformation of the arcs when they are assembled, the fit clearance areas of each structure are different. To ensure uniformity of assembly fit clearance areas, the difference between them should be small. Therefore, the variance of the fit clearance areas between each arc and mirror was used as a measure of their assembly uniformity. Thus, the position of the minimum variance of the fit clearance areas can be considered as the suitable assembly alignment position of the claw framework.

The fit clearance area between an arc and mirror is shown in Figure 13a. Their areas are denoted as *s*_1_, *s*_2_, …, *s*_7_, and *s*_8_. For the calculations, their measured data were converted to polar coordinates. The fit clearance areas can be described as:

(7) $$s_{i}=\frac{1}{2}\int_{\eta }^{\lambda }\left[\varphi_{i2}^{2}(\tau )-\varphi_{i1}^{2}(\tau )\right]d\tau i=1,2,\ldots ,8$$

Based on the above analysis, the issue of the assembly alignment position of eight-section arcs can be regarded as finding the position that minimizes the variance of fit clearance areas between the assembled parts. The variance of the areas can be described as:

(8) $$D(s)=\frac{1}{8}\sum_{i=1}^{8}{\left(s_{i}-E(s)\right)}^{2}$$

where

(9) $$E(s)=\frac{1}{8}\sum_{i=1}^{8}s_{i}$$

*E*(*s*) and *s_i_* can be substituted into the above equation, and the optimization model is:

(10) $$\min\frac{1}{8}\sum_{i=1}^{8}{\left\{\frac{1}{2}\int_{\eta_{i}}^{\lambda_{i}}\left[\varphi_{i2}^{2}(\tau )-\varphi_{i1}^{2}(\tau )\right]d\tau -\frac{1}{16}\sum_{i=1}^{8}\int_{\eta_{i}}^{\lambda_{i}}\left[\varphi_{i2}^{2}(\tau )-\varphi_{i1}^{2}(\tau )\right]d\tau \right\}}^{2}$$

where [*λ_i_*, *η_i_*] (*i*=1, 2, …, 8) represents the angle range of each arc in polar coordinates.

Based on the measured data of the parts, the solution for fit clearance areas can be obtained using the numerical integration method. However, due to the complexity of the objective function in the optimization model; therefore, considering the actual assembly requirements and visual system resolution, this study uses the grid search method to solve the problem. The calculation process is described as follows.

First, the approximate fitting centers were obtained using the least squares fitting method based on the data of the eight-section arcs of the claw framework. As the mirror has high machining accuracy, its outer circle is considered an ideal circle, and its center can be directly calculated using the least squares fitting method. Secondly, a search grid was constructed based on the precision of the measurements. The initial search position of the assembly with the mirror is taken as the approximate fitting center of the eight-section arcs. While the position of the eight-section arcs in the coordinate system remained unchanged, the mirror was translated within the search grid. The variance of the fit clearance areas within the search grid could be calculated, and the suitable assembly alignment position of the part with eight-section arcs was determined by finding the position of the minimum variance of the search point.

**Figure 14:**
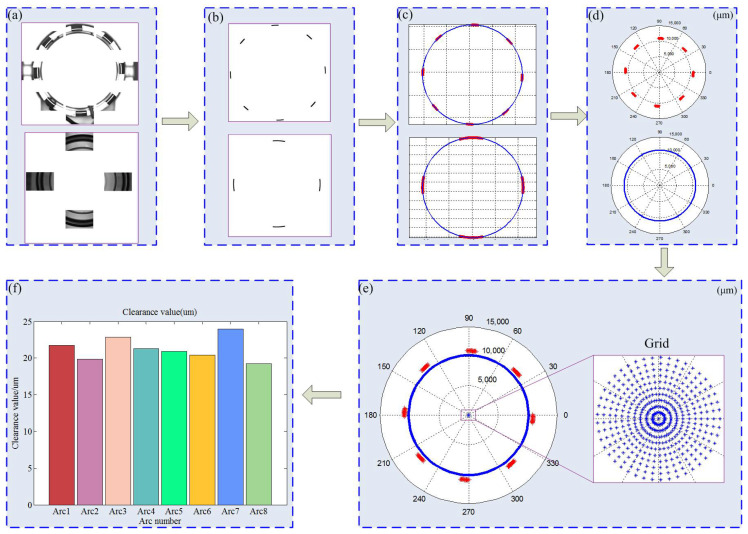
Flowchart of calculating the assembly fit clearance values: (**a**) Experimental image of parts; (**b**) Extract the target edges of the images; (**c**) Calculate centers using the least square fit; (**d**) Convert to polar coordinates; (**e**) Calculate the assembly alignment position based on uniformity assembly using grid search; (**f**) Calculate the mean fit clearance values.

The predictive assembly fit clearance values can be calculated based on experimental images, and a flowchart of this algorithm is shown in Figure 14. Firstly, the experimental images were processed using filtering, edge detection, and ROI extraction techniques to obtain the target edges, as illustrated in Figure 14b. Then, their centers and radii were obtained using the least squares fitting method, as shown in Figure 14c. Subsequently, their target data were translated to the position where the center coordinates were (0, 0) and converted to polar coordinates, as demonstrated in Figure 14d. Second, the origin was set to the initial position, and a search grid was created according to the actual measurement accuracy. The claw framework data was fixed, and the fitting circle of the mirror moved within all search points. Meanwhile, the variance of the fit clearance areas was calculated, and the minimum variance value could be found within the search grid, as depicted in Figure 14e. (Due to the designed clearance between the claw framework and the mirror being 20 μm, the radius for grid searching was set to 20 μm. Given that the camera’s pixel equivalent value was 2.46 μm, the grid search interval was slightly smaller, chosen to be 2 μm.) Finally, using the position of the minimum variance of the fit clearance areas, the mean fit clearance values of the predictive assembly can be calculated, as shown in Figure 14f. These fit clearance values can be used to determine whether a part satisfies the assembly requirements.

### 4.3. Inspection Experiment

To demonstrate the effectiveness of the proposed method, we conducted an inspection on three claw frameworks (1#, 2#, and 3#) and another assembly part of a mirror with a diameter of C μm. Images of the claw frameworks and the mirror were obtained using the visual model of the assembly system. In the inspection process, we first calculated the grouping diameters of the arcs in the opposite direction of the claw frameworks. Then, we computed the mean values of the fit clearance between each arc of the claw framework and the mirror for predictive assembly.

**Figure 15:**
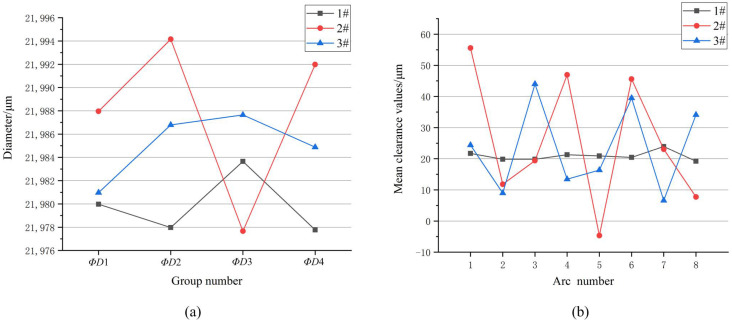
Grouping diameters and fit clearance values: (**a**) Diameter results for claw framework 1#, 2#, 3# (**b**) Predictive assembly means fit clearance values for 1#, 2#, 3#.

In Figure 15a, we present the grouping diameter results of the eight-section arcs, while Figure 15b shows the mean fit clearance values of the claw frameworks with the mirror. The grouping diameters of the arcs for the claw framework should fall within the range of *A* to *B* μm, according to the design size, to meet the processing requirements. Similarly, the assembly fit clearance value range with the mirror should be between 10 μm and 40 μm to ensure compliance with assembly requirements. Our analysis reveals that the diameters of the three parts presented in Figure 15a fall within the range of *A*-*B* μm, satisfying the processing requirements. However, as demonstrated by Figure 15b, only part 1# meets the assembly requirements, with a fit clearance value that falls within the permissible range. In contrast, the fit clearance values of parts 2# and 3# exceed the threshold of 40 μm. Therefore, only the part 1# is deemed qualified for assembly purposes.

The inspection of arc structures based solely on the design size can sometimes result in false detections. However, by considering the assembly fit clearance values simultaneously, it is possible to determine whether a part meets the precision assembly requirements. The proposed method, which utilizes fit clearance values, provides a suitable approach for evaluating the suitability of parts for assembly purposes. This approach offers an effective means of ensuring compliance with the assembly requirements. Using this method, false inspections can be avoided, and the accuracy and reliability of the inspection process can be improved in precision assembly.

## 5. Conclusions

This study focuses on the inspection of parts with micro multi-section arcs in the context of the precision assembly of an optic-mechanical device. To address the challenges associated with image ROI extraction, we propose a feature point extraction method that combines CAD-based techniques with connected region detection. By establishing the geometric relationship between feature points of images with micro multi-section arc structures, using the CAD model, we can obtain an approximate location of feature points that cannot be extracted directly. This information serves as prior information and is subsequently refined using the connected region detection method. Subsequently, based on feature points, the CAD model is used again to obtain the ROI. Regarding the inspection of parts, this study proposes a novel method based on the assembly requirements of the fit clearance values. Specifically, we present a methodology based on the principle of uniformity to determine the assembly alignment position. We used the variance of the fit clearance areas as a measure of the assembly uniformity to obtain a suitable assembly alignment position. Finally, experimental testing was performed to demonstrate the validity of the proposed method. The results indicate that the proposed method provides a suitable and accurate solution for the inspection of micro multi-section arcs.

The algorithm for ROI extraction based on the CAD model gives another means for image processing of mechanical parts. The assembly alignment position based on uniformity assembly also shows a novel method for the assembly position of multi-section arcs in precision assembly. The method using fit clearance provides a suitable inspection technique in precision assembly.

## Figures and Tables

**Table 1:** Origin coordinates of the ROI.

NO.	(*X*, *Y*)	(*X*′,*Y*′)
1	$\left(x_{1}-\Delta x,\;y_{1}\right)$	$\left(x_{1}+\Delta x,\;y_{1}+\Delta y\right)$
2	$\left(x_{2}-\frac{\sqrt{2}}{2}(\Delta x+d),\;y_{2}-\frac{\sqrt{2}}{2}(\Delta x-d)\right)$	$\left(x_{2}+\frac{\sqrt{2}}{2}(\Delta x-d),\;y_{2}+\frac{\sqrt{2}}{2}(\Delta x+d)\right)$
3	$\left(x_{3}-\Delta y,\;y_{3}-\Delta x\right)$	$\left(x_{3},\;y_{3}+\Delta x\right)$
4	$\left(x_{4}-\frac{\sqrt{2}}{2}(\Delta x+d),\;y_{4}-\frac{\sqrt{2}}{2}(\Delta x+d)\right)$	$\left(x_{4}+\frac{\sqrt{2}}{2}(\Delta x-d),\;y_{4}+\frac{\sqrt{2}}{2}(\Delta x-d)\right)$
5	$\left(x_{5}-\Delta x,\;y_{5}-\Delta y\right)$	$\left(x_{5}+\Delta x,\;y_{5}\right)$
6	$\left(x_{6}-\frac{\sqrt{2}}{2}(\Delta x-d),\;y_{6}-\frac{\sqrt{2}}{2}(\Delta x+d)\right)$	$\left(x_{6}+\frac{\sqrt{2}}{2}(\Delta x+d),\;y_{6}+\frac{\sqrt{2}}{2}(\Delta x-d)\right)$
7	$\left(x_{7},\;y_{7}-\Delta x\right)$	$\left(x_{7}+\Delta y,\;y_{7}+\Delta x\right)$
8	$\left(x_{8}-\frac{\sqrt{2}}{2}(\Delta x-d),\;y_{8}-\frac{\sqrt{2}}{2}(\Delta x-d)\right)$	$\left(x_{8}+\frac{\sqrt{2}}{2}(\Delta x+d),\;y_{8}+\frac{\sqrt{2}}{2}(\Delta x+d)\right)$

## Data Availability

Data underlying the results presented in this paper are not publicly available at this time but may be obtained from the authors upon reasonable request.
